# SMAD4 loss enables EGF, TGFβ1 and S100A8/A9 induced activation of critical pathways to invasion in human pancreatic adenocarcinoma cells

**DOI:** 10.18632/oncotarget.12068

**Published:** 2016-09-16

**Authors:** Stefania Moz, Daniela Basso, Dania Bozzato, Paola Galozzi, Filippo Navaglia, Ola H. Negm, Giorgio Arrigoni, Carlo-Federico Zambon, Andrea Padoan, Paddy Tighe, Ian Todd, Cinzia Franchin, Sergio Pedrazzoli, Leonardo Punzi, Mario Plebani

**Affiliations:** ^1^ University of Padova, Laboratory Medicine, Department of Medicine - DIMED, Padova, Italy; ^2^ University of Padova, Rheumatology Unit, Department of Medicine - DIMED, Padova, Italy; ^3^ University of Nottingham, School of Life Sciences, Queen's Medical Centre, Nottingham, UK; ^4^ Mansoura University, Medical Microbiology and Immunology Department, Faculty of Medicine, Mansoura City, Egypt; ^5^ University of Padova, Department of Biomedical Sciences, Padova, Italy; ^6^ Proteomics Center, University of Padova and Azienda Ospedaliera di Padova, Padova, Italy; ^7^ Associazione Wirsung-onlus, Padova, Italy

**Keywords:** pancreatic cancer, SMAD4, signalling, epidermal growth factor, transforming growth factor β1

## Abstract

Epidermal Growth Factor (EGF) receptor overexpression, KRAS, TP53, CDKN2A and SMAD4 mutations characterize pancreatic ductal adenocarcinoma. This mutational landscape might influence cancer cells response to EGF, Transforming Growth Factor β1 (TGFβ1) and stromal inflammatory calcium binding proteins S100A8/A9. We investigated whether chronic exposure to EGF modifies in a SMAD4-dependent manner pancreatic cancer cell signalling, proliferation and invasion in response to EGF, TGFβ1 and S100A8/A9. BxPC3, homozigously deleted (HD) for SMAD4, and BxPC3-SMAD4+ cells were or not stimulated with EGF (100 ng/mL) for three days. EGF pre-treated and non pretreated cells were stimulated with a single dose of EGF (100 ng/mL), TGFβ1 (0,02 ng/mL), S100A8/A9 (10 nM). Signalling pathways (Reverse Phase Protein Array and western blot), cell migration (Matrigel) and cell proliferation (XTT) were evaluated. SMAD4 HD constitutively activated ERK and Wnt/β-catenin, while inhibiting PI3K/AKT pathways. These effects were antagonized by chronic EGF, which increased p-BAD (anti-apoptotic) in response to combined TGFβ1 and S100A8/A9 stimulation. SMAD4 HD underlied the inhibition of NF-κB and PI3K/AKT in response to TGFβ1 and S100A8/A9, which also induced cell migration. Chronic EGF exposure enhanced cell migration of both BxPC3 and BxPC3-SMAD4+, rendering the cells less sensitive to the other inflammatory stimuli. In conclusion, SMAD4 HD is associated with the constitutive activation of the ERK and Wnt/β-catenin signalling pathways, and favors the EGF-induced activation of multiple signalling pathways critical to cancer proliferation and invasion. TGFβ1 and S100A8/A9 mainly inhibit NF-κB and PI3K/AKT pathways and, when combined, sinergize with EGF in enhancing anti-apoptotic p-BAD in a SMAD4-dependent manner.

## INTRODUCTION

Pancreatic ductal adenocarcinoma (PDAC), the fourth leading cause of cancer-related deaths in the US and the seventh worldwide [1, 2], is characterized by a complex mutational landscape and significant inter-tumoral genetic heterogeneity. Based on the results of whole genome sequencing and copy number variation analysis of 100 PDAC, Waddell et al. [3] suggested to classify PDAC into four subtypes (stable, locally rearranged, scattered and unstable), the patterns of chromosome variation being recognized as an important mutational mechanism in pancreatic carcinogenesis. In addition to numerous genes mutated at low prevalence, the mutations of four genes (KRAS, TP53, SMAD4, CDKN2A) characterize PDAC. Highly frequent early events in PDAC are activating mutations of the KRAS oncogene and inactivating mutations of the tumor suppressor gene TP53 (more than 90% and up to 70% of the cases, respectively), whereas late events, found in 30 to 50% of cases, are inactivation of the tumor suppressors CDKN2A and SMAD4 due to homozygous deletion and/or a combination of structural variation events with deleterious point mutations [4].

On the basis of results obtained in transgenic mouse models, KRAS mutation was found to be necessary for, but not sufficient to cause, PDAC, the further onset and progression of which is dependent on KRAS mutation combined with inactivation of tumor suppressor genes, TP53 or SMAD4 in particular [5, 6]. Data supporting the role of TP53 and SMAD4 inactivation in human PDAC carcinogenesis derive mainly from the study of PanINs precursor lesions [7]. Most of the studies conducted to investigate whether these mutations play a role in tumor progression provide evidence that SMAD4 deletion is a negative prognostic factor [8–13], although recently Dal Molin et al. [14] failed to demonstrate that somatic KRAS, TP53, SMAD4 and CDKN2A mutations impact on PDAC survival in the very long-term. This discrepancy may depend on the fact that PDAC behaviour not only depends on the genetic derangement of cancer cells, but is also the result of a complex interplay between genetically altered cancer cells and their surrounding microenvironment. PDAC has a highly dispersed growth pattern, with wide-set tumour glands in extensive desmoplastic stroma that encompasses inflammatory and stellate cells, and fibroblasts [15, 16]. Stromal and cancer cells influence each other through contact dependent and independent mechanisms, the latter being mediated by the release of soluble factors, which include cytokines, chemokines, growth factors and inflammatory molecules. In this scenario the epidermal growth factor (EGF), the transforming growth factor β1 (TGFβ1) and the inflammatory molecules S100A8 and S100A9 seem particularly relevant [17–19]. Cancer cells not only release EGF, but they also overexpress the EGF receptor (EGFR), which is recognized as the initial, indispensable molecular alteration in pancreatic carcinogenesis. As a general rule, EGF determines the activation of the MAPK/ERK and PI3K/AKT pathways [20]. TGFβ1, produced by cancer and stromal cells, is primarily involved in the desmoplastic reaction, but it may also support or antagonize cancer cell survival and dissemination [18, 21]. These dual TGFβ1 effects depend on the status of cancer cells and on the intracellular signalling events evoked upon the binding of its receptors. TGFβ1 signalling has been previously reported as one of the 12 core signalling pathways altered in PDAC [22], and recently it was identified as part of a core transcriptional gene program including also PDGF, VEGF, Ras, integrin, PI3K/AKT and Wnt signalling, which increased expression characterizes the most lethal PDAC squamous subtype [23]. TGFβ1 activates SMAD-dependent canonical and SMAD-independent non-canonical signal pathways. Canonical TGFβ signalling refers to the receptor-mediated carboxy terminal phosphorylation of the receptor-regulated SMAD2/3 (R-Smads) promoting binding to the common mediator SMAD4 (Co-Smad), and the further translocation of the heterocomplex in the nucleus thus initiating the transcription of extracellular matrix proteins, such as fibronectin, collagen and proteoglycans [24]. The canonical TGFβ signalling pathway is regulated by the inhibitor Smads (I-Smads) SMAD6 and SMAD7, which act by inhibiting R-Smads by competitive binding with SMAD4 [24]. Non-canonical TGFβ signalling activates different pathways like JNK, MAPK/ERK, PI3K/AKT and NF-κB. Multiple mechanisms are implicated in non-canonical TGFβ signalling, including the imbalance between the TGFβ1 receptors, TβRI and TβRII, and the tyrosine phosphorylation of the cytoplasmic tail of TβRII in the activation of ERK, MAPK or JNK. It is not yet completely understood the mechanism underlying PI3K/AKT and NF-κB pathways activation [25–27]. The activation of non-canonical MAPK/ERK, JNK or AKT signalling pathways might prevent canonical TGFβ signalling because they regulate stability, activity and nuclear transport of R-Smads through the phosphorylation of the serine/threonine residues of the linker region [24]. Since SMAD4 plays a crucial role in the canonical TGFβ signalling pathway, SMAD4 gene deletion should necessarily determine an altered cellular response to TGFβ1 stimulation. As is the case for TGFβ1, the calcium binding proteins S100A8/A9 are dual faced molecules that are not only involved in PDAC growth and dissemination, but can also form complexes with TGFβ1 thus concurring in further enhancing the spectrum of the effects evoked in cancer cells by this cytokine [28]. The expression of S100A8/A9 is also intimately linked with SMAD4 status: it is prevalent in stromal cells when cancer cells express SMAD4, whereas in SMAD4 homozygous deletion cancer cells independently acquire the ability to express S100A8/A9 [29–31]. Through the engagement of RAGE and TLR4, S100A8/A9 receptors activate several intracellular signalling pathways, such as NF-κB, MAPK/ERK and JNK [32].

Due to their relevance in cancer biology, therapeutic targeting of EGF, but also of PI3K/AKT/mTOR pathways, appears a promising treatment strategy [33, 34]. However, any benefit from targeted therapy for PDAC in clinical practice has, so far, only been minor in terms of disease free survival and overall survival [33, 35]. One of the reasons for treatment failure might be resistance to targeted therapies developed by tumor cells, since they not only accumulate genetic defects during tumor progression but they also adapt to a changing microenvironment. Adaptation might lead to activation of alternative growth factor receptor pathways or it may be the result of constitutively activated downstream intracellular signals, being an increased expression of the EGF receptor signalling pathway a signature of the most aggressive PDAC subtype [23].

The present “*in vitro*” study was made in order to assess whether the exposure of cancer cells to EGF in the tumor microenvironment modifies pancreatic cancer cell signalling, proliferation and invasion in response to EGF itself, TGFβ1 and S100A8/A9 singly or combined and whether SMAD4 has a part in this scenario. By using a microarray system that enables us to simultaneously analyse multiple signalling pathways, we demonstrated that in cells without SMAD4 expression, TGFβ1 and S100A8/A9 inhibit NF-κB and PI3K/AKT pathways. Chronic EGF stimulation enhanced cell invasion, possibly through MMP9 expression, while flattening the overall cell signalling response to the studied stimuli in a SMAD4-independent manner.

## RESULTS

### Characterization of the cellular model

BxPC3 cells, HD for SMAD4, and BxPC3-SMAD4+, a SMAD4 transfected BxPC3 cell clone, were used in this study. By RT-PCR BxPC3-SMAD4+ cells were found to express SMAD4 mRNA (mean C_T_ ± SD of 4 independent experiments each made in duplicate = 20.81 ± 0.56) at levels higher than those of the SMAD4+ cell lines MiaPaCa2 (24.70 ± 0.39) and Panc1 (25.25 ± 0.46). SMAD4 expression was not detected in BxPC3 cells (C_T_ always above 30). Supplementary Figure 1 shows the melting curves of a representative experiment. Smad4 protein was evidenced by western blot analysis in the cell lines used as positive controls (MiaPaCa2 and Panc1), but not in BxPC3 nor in BxPC3-SMAD4+ cells (Supplementary Figure 1). To assess whether SMAD4 mRNA expression has an impact on the cellular proteome, the expression of BxPC3 and BxPC3-SMAD4+ cellular proteins was compared by means of SILAC experiments. Based on the results of two independent experiments, a total of 1476 and 1102 proteins were identified by means of Proteome Discoverer software. The results of the two experiments were matched and averaged, this resulting in a total of 1002 proteins reported in Supplementary Table 1. A significant differential expression was considered when the ratio between BxPC3 and BxPC3-SMAD4+ for any protein was below 0.67 (underexpressed in BxPC3, *n* = 26 proteins) or above 1.5 (overexpressed in BxPC3, *n* = 89 proteins).

### SMAD4 HD enables the constitutive and EGF-induced activation of multiple signalling pathways

The effects of SMAD4 on cell signalling in response to chronic EGF stimulation were first investigated. To obtain a comprehensive overview of intracellular signalling pathways, RPPA analysis was performed comparing BxPC3 and BxPC3-SMAD4+, cultured in the absence or in the presence of 100 ng/mL EGF for three days. RPPA results (Table 1) showed that SMAD4 expression impacts on PI3K/AKT, ERK and NF-κB pathways, while EGF and SMAD4 interact and impact on MAP kinase, ERK and apoptosis pathways. RPPA data were confirmed by the western blot analyses of the representative targets, p-AKT (Thr^308^ and Ser^473^), and p-Iκ-Bα (Ser^32^) (Supplementary Figure 2). The mTOR and Wnt/β-catenin pathways were also investigated in the above-described conditions, by means of p-mTOR (Ser^2481^ and Ser^2448^) and p-β-catenin (Ser^33/37^/Thr^41^) analyses (Supplementary Figure 2). Independently from SMAD4 expression, the exposure of cells to EGF chronic stimulation caused both increased (Ser^2448^) and decreased mTOR (Ser^2481^) phosphorylation, while p-β-catenin was induced in BxPC3 but not in BxPC3-SMAD4+ cells.

**Table 1:** Reverse phase protein array (RPPA) data obtained from unstimulated and from EGF chronically stimulated pancreatic cancer cells expressing (BxPC3-SMAD4+) or not (BxPC3) SMAD4

TNF receptor 1 pathway	Model		EGF	SMAD4	EGF and SMAD4
	***F* value**	***p* value**	***p* value**	***p* value**	***p* value**
TRAF2	0.45	0.7241	0.3106	0.7728	0.7721
p-RIP2 (Ser^176^)	2.14	0.1739	0.1330	0.1304	0.4062
A20/TNFAIP3	0.61	0.6256	0.3606	0.3739	0.9184
**NF-kB pathway**	**Model**		**EGF**	**SMAD4**	**EGF and SMAD4**
	***F* value**	***p* value**	***p* value**	***p* value**	***p* value**
p-IKKα/β (Ser^176^/Ser^177^)	5.56	**0.0233**	0.8008	**0.0039**	0.4736
IKKα	1.07	0.4154	0.6599	0.1222	0.9356
IKKβ	3.16	0.0858	0.1434	0.0310	0.9011
p-NF-kB p65 (Ser^536^)	3.48	0.0703	0.3467	0.0280	0.1700
**PI3K/AKT pathway**	**Model**		**EGF**	**SMAD4**	**EGF and SMAD4**
	***F* value**	***p* value**	***p* value**	***p* value**	***p* value**
SHIP2	1.56	0.2736	0.0868	0.9416	0.3810
p-eNOS (Ser^1177^)	1.69	0.2455	0.6894	0.0603	0.7372
p-AKT (Thr^308^)	9.80	**0.0047**	0.3509	**0.0007**	0.6495
p-AKT (Ser^473^)	1.53	0.2806	0.5316	0.0759	0.9763
PI3K p85	4.62	**0.0371**	0.5307	**0.0064**	0.9064
PI3K p100a	1.95	0.2005	0.6238	0.0535	0.5140
p-GSK3b (Ser^9^)	3.97	0.0527	0.3360	0.0109	0.9584
p-PTEN (Ser^380^)	4.65	**0.0366**	0.3266	0.0247	0.0515
**MAP kinase pathway**	**Model**		**EGF**	**SMAD4**	**EGF and SMAD4**
	***F* value**	***p* value**	***p* value**	***p* value**	***p* value**
p-HSP27 (Ser^82^)	9.37	**0.0054**	0.4823	0.1388	**0.0011**
p-p38 MAPK (Thr^180^/Thr^182^)	2.41	0.1426	0.6049	0.1035	0.0961
**c-Jun pathway**	**Model**		**EGF**	**SMAD4**	**EGF and SMAD4**
	***F* value**	***p* value**	***p* value**	***p* value**	***p* value**
p-SAPK/JNK (Thr^183^/Tyr^185^)	1.49	0.2901	0.3928	0.1014	0.6526
MKK7	7.29	**0.0112**	0.1370	0.0099	0.0233
**ERK pathway**	**Model**		**EGF**	**SMAD4**	**EGF and SMAD4**
	***F* value**	***p* value**	***p* value**	***p* value**	***p* value**
MEK1/2	0.60	0.6344	0.5322	0.3011	0.7145
p-MEK1/2 (Ser^217^/Ser^221^)	2.27	0.1572	0.1269	0.1359	0.3119
ERK1/2	12.91	**0.0020**	0.3133	0.0011	0.0076
p-ERK1/2 (Thr^202^/Tyr^204^)	42.18	**< 0.0001**	0.3334	0.0025	< 0.0001
**SRC/JAK/STAT pathway**	**Model**		**EGF**	**SMAD4**	**EGF and SMAD4**
	***F* value**	***p* value**	***p* value**	***p* value**	***p* value**
p-STAT3 (Tyr^705^)	4.40	0.0417	0.0122	0.6376	0.1482
SOCS3	1.96	0.1990	0.6011	0.1415	0.1261
**IL-1b pathway**	**Model**		**EGF**	**SMAD4**	**EGF and SMAD4**
	***F* value**	***p* value**	***p* value**	***p* value**	***p* value**
TRAF6	2.84	0.1060	0.9780	0.0595	0.0909
p-TAK1 (Ser^412^)	3.50	0.0696	0.8759	0.0386	0.0702
**Inflammasome pathway**	**Model**		**EGF**	**SMAD4**	**EGF and SMAD4**
	***F* value**	***p* value**	***p* value**	***p* value**	***p* value**
p-STAT1 (Tyr^701^)	3.75	0.0599	0.1726	0.0449	0.1036
**Apoptosis pathway**	**Model**		**EGF**	**SMAD4**	**EGF and SMAD4**
	***F* value**	***p* value**	***p* value**	***p* value**	***p* value**
BCL-2	1.24	0.3582	0.1910	0.8625	0.2361
p-BAD (Ser^136^)	8.60	**0.0069**	0.4614	0.1038	**0.0016**

The cells were cultured for three days in the absence or in the presence of 100 ng/mL EGF which was added daily. For any target the percentage changes in fluorescence intensity relative to untreated BxPC3 were calculated from three independent experiments. EGF pre-treated and non pre-treated BxPC3 and BxPC3-SMAD4+ cells were compared with each other by the analysis of variance (ANOVA) including in the model the effects of EGF, SMAD4 and of their interaction.

### TGFβ1 and S100A8/A9 inhibits NF-κB and AKT pathways and concurs with EGF in inhibiting apoptosis

**Figure 1:**
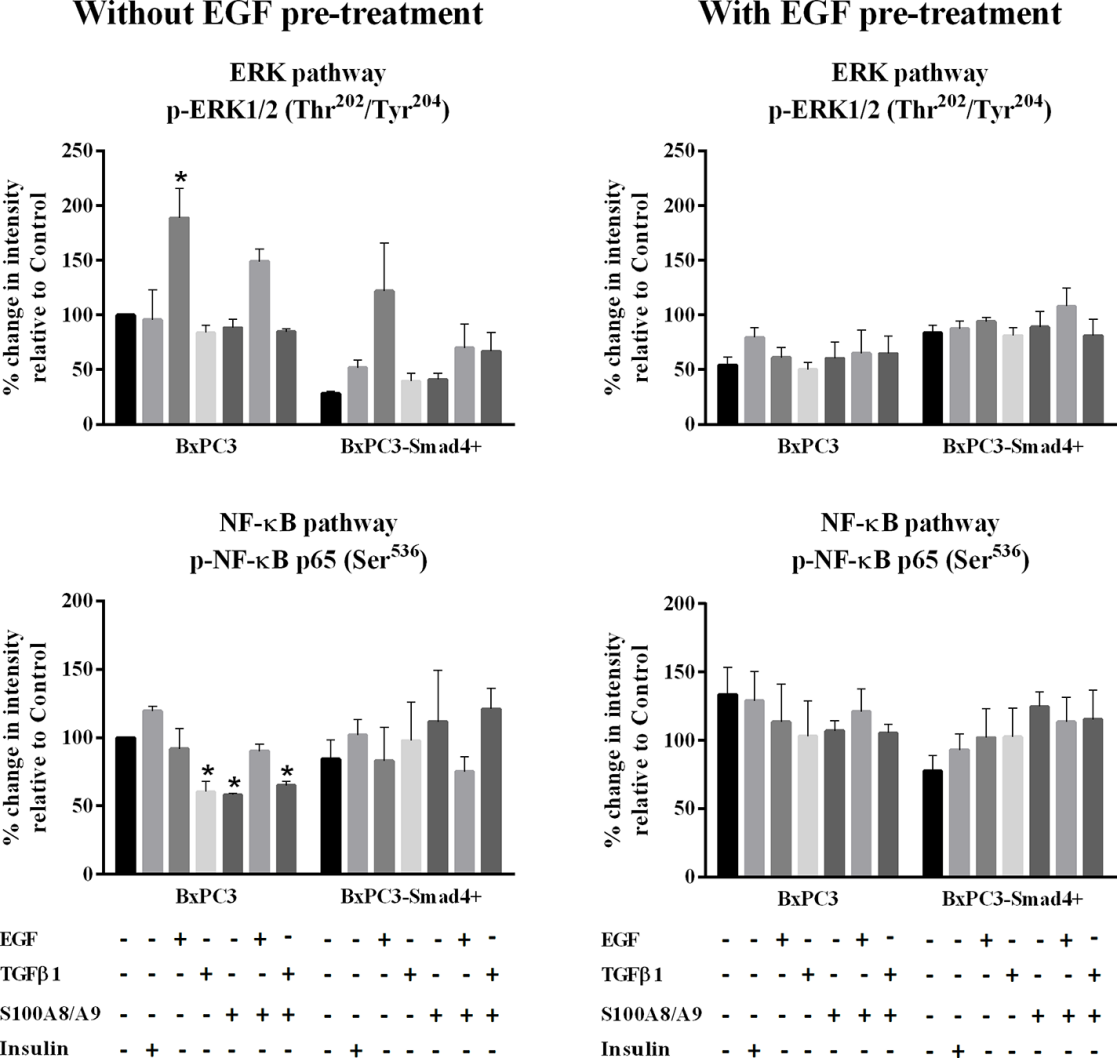
ERK and NF-κB pathways Representative data from Reverse Phase Protein Array (RPPA) analysis obtained from unstimulated and from EGF chronically stimulated pancreatic cancer cells expressing (BxPC3-SMAD4+) or not (BxPC3) SMAD4. The cells were cultured for three days in the absence or in the presence of 100 ng/mL EGF which was added daily. At the fourth day the cells were stimulated with EGF 100 ng/mL, TGFβ1 0.02 ng/mL and S100A8/A9 10 nM alone or combined, and with insulin 50 mU or they were left untreated. The percentage changes in fluorescence intensity relative to BxPC3 without EGF pre-treatment (control) were calculated and reported as mean ± SD (three independent experiments). * = statistically significant with respect to control.

**Figure 2:**
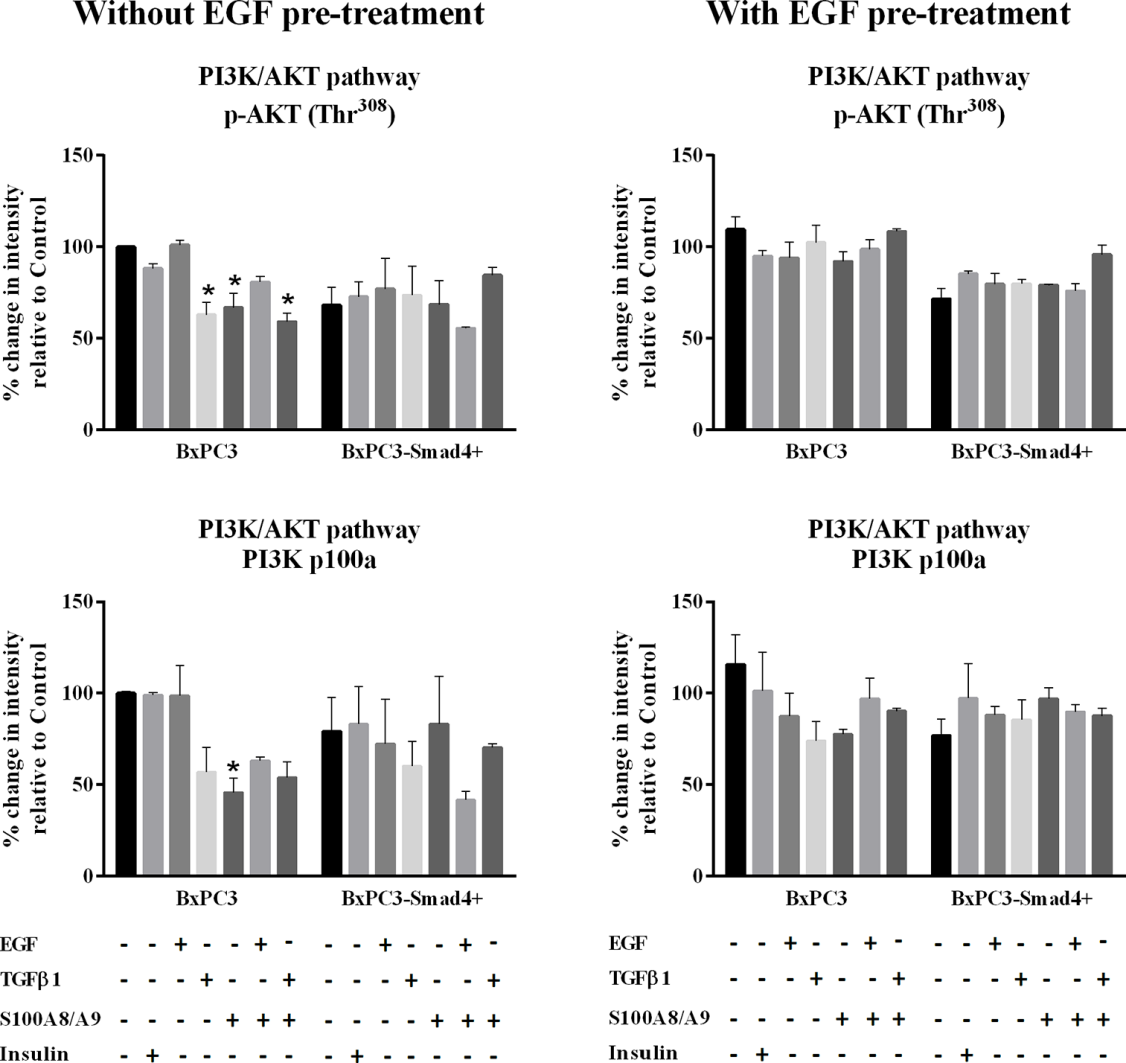
PI3K/AKT pathway Representative data from Reverse Phase Protein Array (RPPA) analysis obtained from unstimulated and from EGF chronically stimulated pancreatic cancer cells expressing (BxPC3-SMAD4+) or not (BxPC3) SMAD4. The cells were cultured for three days in the absence or in the presence of 100 ng/mL EGF which was added daily. At the fourth day the cells were stimulated with EGF 100 ng/mL, TGFβ1 0.02 ng/mL and S100A8/A9 10 nM alone or combined, and with insulin 50 mU or they were left untreated. The percentage changes in fluorescence intensity relative to BxPC3 without EGF pre-treatment (control) were calculated and reported as mean ± SD (three independent experiments). * = statistically significant with respect to control.

**Figure 3:**
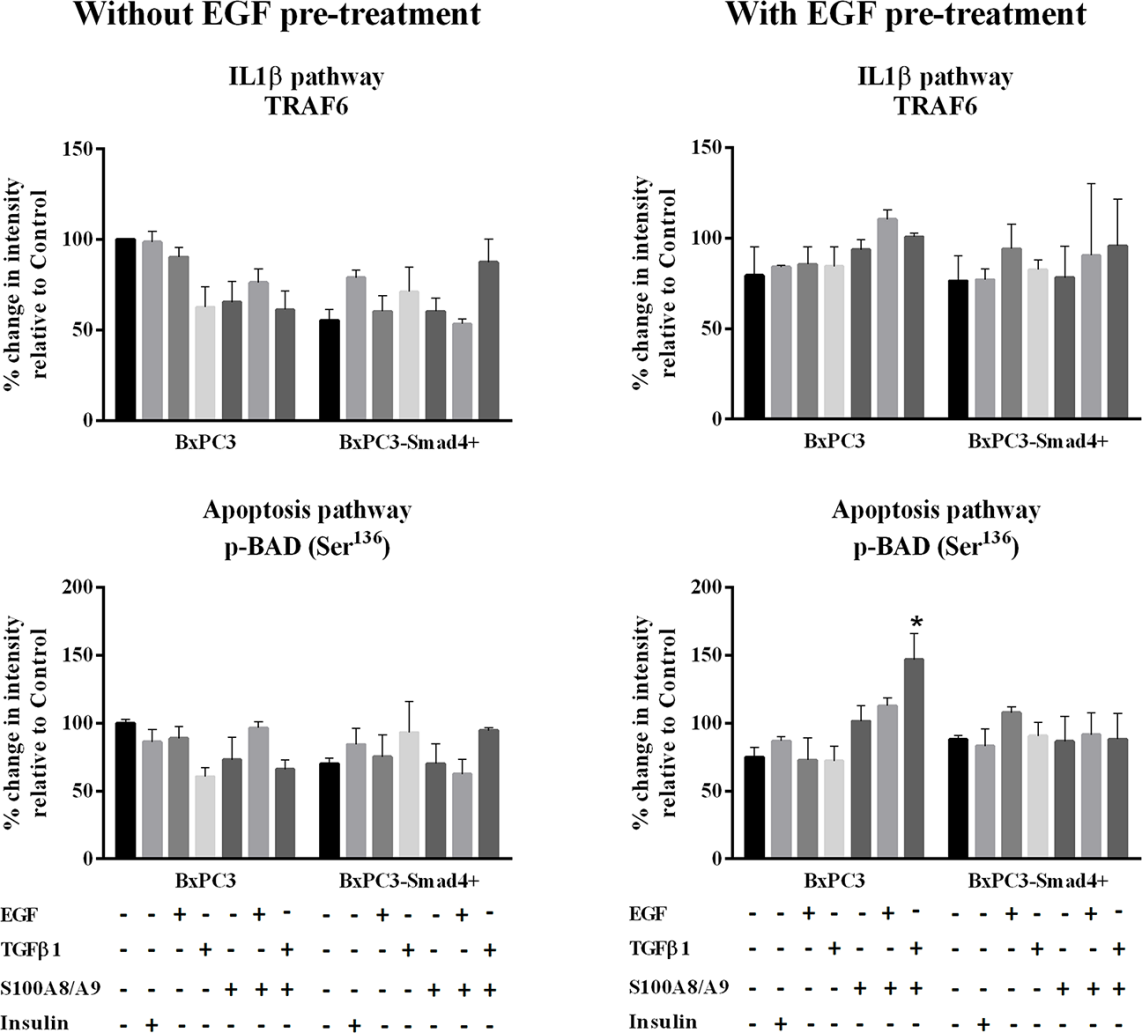
IL-1β and apoptosis pathways Representative data from Reverse Phase Protein Array (RPPA) analysis obtained from unstimulated and from EGF chronically stimulated pancreatic cancer cells expressing (BxPC3-SMAD4+) or not (BxPC3) SMAD4. The cells were cultured for three days in the absence or in the presence of 100 ng/mL EGF which was added daily. At the fourth day the cells were stimulated with EGF 100 ng/mL, TGFβ1 0.02 ng/mL and S100A8/A9 10 nM alone or combined, and with insulin 50 mU or they were left untreated. The percentage changes in fluorescence intensity relative to BxPC3 without EGF pre-treatment (control) were calculated and reported as mean ± SD (three independent experiments). * = statistically significant with respect to control.

**Figure 4:**
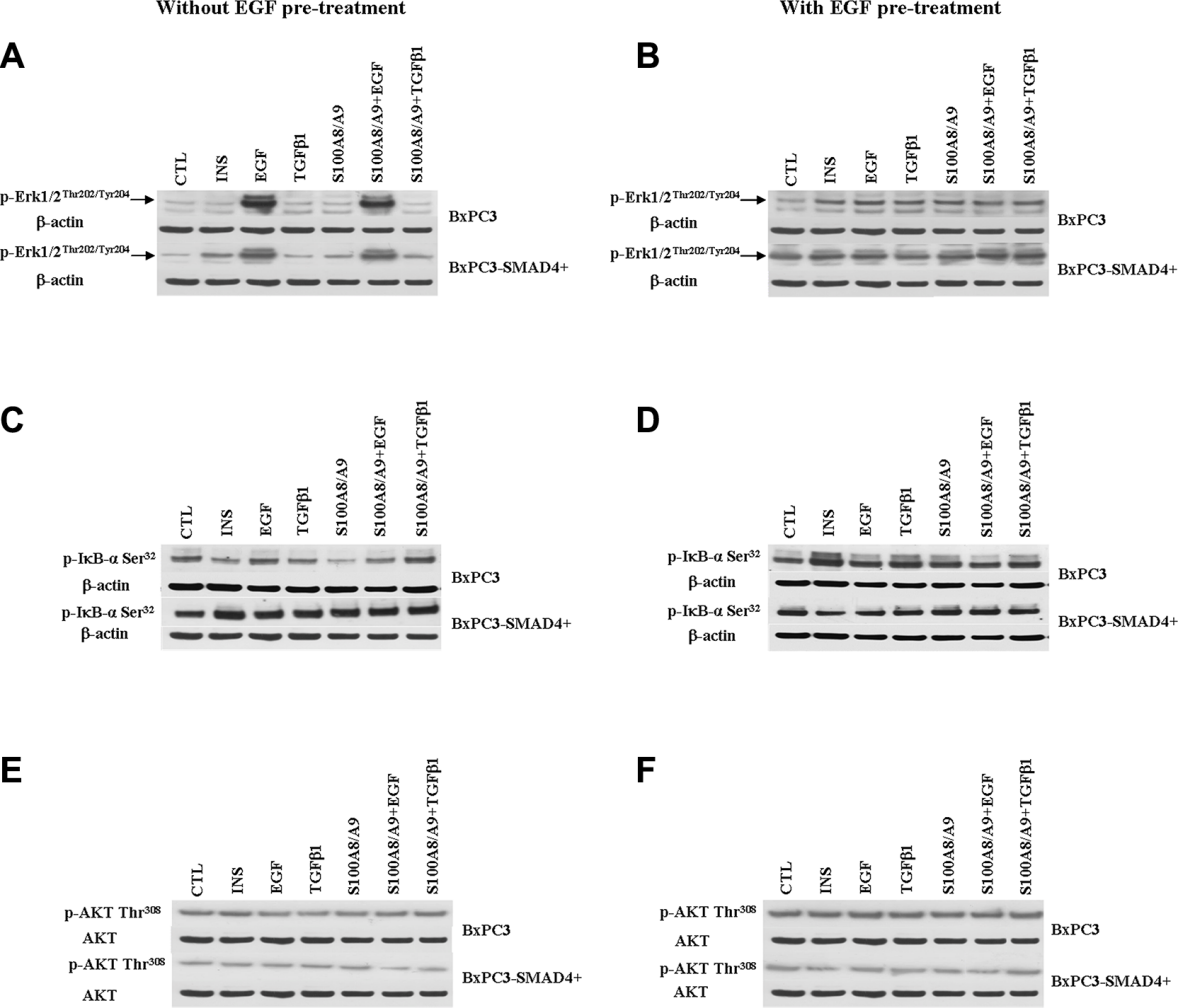
Western blot analyses obtained from pancreatic cancer cells expressing (BxPC3-SMAD4+) or not (BxPC3) SMAD4 and subjected to insulin (INS), EGF, TGFβ1 and S100A8/A9 stimulation in the absence or in the presence of chronic EGF exposure Representative targets of the ERK (panels **A** and **B**), NF-ĸB (panels **C** and **D**), PI3K/AKT (panels **E** and **F**) pathways are shown.

**Figure 5:**
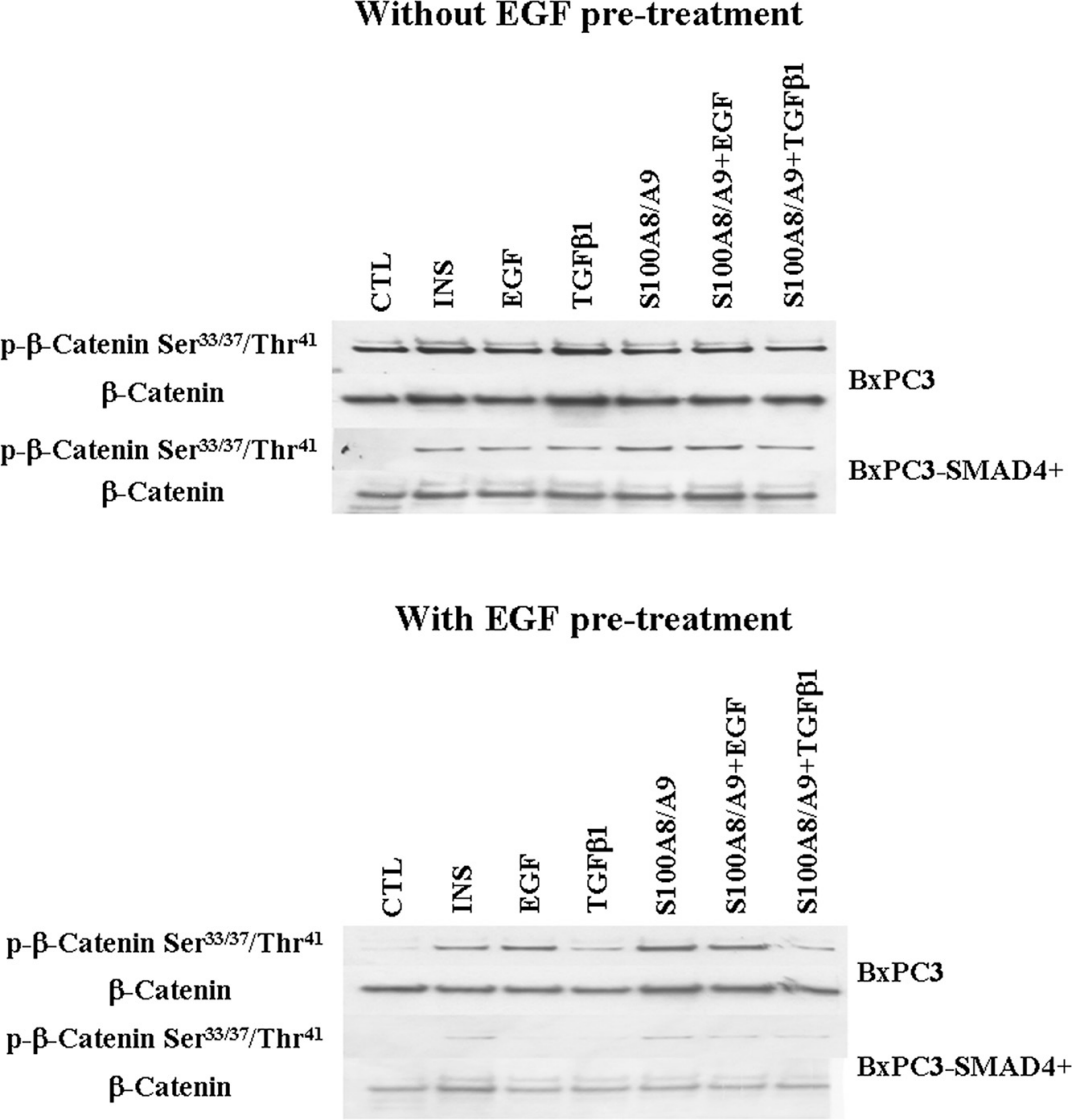
Wnt/β-catenin pathway Western blot analyses of nuclear p-β-catenin and of β-catenin obtained from pancreatic cancer cells expressing (BxPC3-SMAD4+) or not (BxPC3) SMAD4 and subjected to insulin (INS), EGF, TGFβ1 and S100A8/A9 stimulation in the absence or in the presence of chronic EGF exposure.

The EGF-evoked cancer cell response might have depended not only on the mutational status of cancer cells, but also on the interactions between EGF and other stimuli, which are likely to occur in the tumor microenvironment. To investigate the effects of these interactions on cell signalling, RPPA experiments were performed considering BxPC3 and BxPC3-SMAD4+ cells subjected or not to chronic EGF pre-treatment, and acutely stimulated with EGF, TGFβ1 and S100A8/A9 alone or combined. The overall RPPA results are reported in Table 2. Representative targets of the most affected pathways are shown in Figure 1 (ERK and NF-κB), Figure 2 (PI3K/AKT) and Figure 3 (IL1β and apoptosis). EGF, as expected, induced ERK phosphorylation in both BxPC3-SMAD4+ and BxPC3 cells. On chronic exposure of cells to EGF pre-treatment, EGF in a single dose did not induce further ERK phosphorylation, as confirmed by western blot (Figure 4). In BxPC3 cells, TGFβ1 and S100A8/A9, alone or combined, inhibited NF-κB, AKT and IL-1β pathways and reduced BAD phosphorylation, thus favouring its pro-apoptotic effect; all these effects were abolished by chronic EGF pre-treatment. In SMAD4 expressing BxPC3-SMAD4+ cells, combined S100A8/A9 and EGF treatment inhibited NF-κB [p-IKKα/β (Ser^176^/Ser^177^), IKKα, IKKβ, p-NF-κB p65 (Ser^536^)] and AKT [p-AKT (Thr^308^), p-AKT (Ser^473^), PI3K p100a, PI3K p85] pathways. These effects were reversed by EGF pre-treatment. Smad2 and Smad3 phosphorylation in whole cell lysates were also evaluated in the above described conditions and results are shown in Supplementary Figure 3. Both proteins were expressed in BxPC3 and BxPC3-SMAD4+ cells and their expression was independent from stimuli. Smad2 and Smad3 phosphorylation was never found. The same results were obtained by the analysis of nuclear extracts (not shown). The mTORC pathway was also investigated by western blot (Supplementary Figure 3). All studied molecules induced the phosphorylation of mTOR, mainly at the Ser^2481^ site, and to a greater extent in BxPC3 than in BxPC3-SMAD4+ cells. The Wnt/β-catenin pathway was studied by the analysis of the nuclear accumulation of p-β-catenin (Ser^35/37^/Thr^41^) (Figure 5), which was induced by all stimuli in both BxPC3 and BxPC3-SMAD4+ cells. Chronic exposure to EGF abolished p-β-catenin nuclear accumulation in BxPC3 and caused a reduced p-β-catenin nuclear accumulation after stimuli in BxPC3-SMAD4+, not in BxPC3.

**Table 2:** Reverse phase protein array (RPPA) data obtained from unstimulated and from EGF chronically stimulated pancreatic cancer cells expressing (BxPC3-SMAD4+) or not (BxPC3) SMAD4

	BxPC3		BxPC3-SMAD4+		Pre-treated BxPC3		Pre-treated BxPC3-SMAD4+	
**TNF receptor 1 pathway**	***F* value**	***p* value**	***F* value**	***p* value**	***F* value**	***p* value**	***F* value**	***p* value**
A20/TNFAIP3	0.30	0.9258	0.67	0.6788	0.57	0.7481	1.29	0.3225
TRAF2	2.54	0.0710	0.80	0.5862	1.03	0.4476	1.33	0.3076
p-RIP2 (Ser^176^)	1.42	0.2755	0.82	0.5723	0.36	0.8945	1.18	0.3703
**NF-kB pathway**	***F* value**	***p* value**	***F* value**	***p* value**	***F* value**	***p* value**	***F* value**	***p* value**
p-IKKα/β (Ser^176^/Ser^177^)	6.52	**0.0019**	1.72	0.1893	1.46	0.2618	2.18	0.1071
IKKα	5.57	**0.0039**	0.42	0.8569	0.79	0.5945	0.24	0.9566
p-NF-kB p65(Ser^536^)	11.29	**0.0001**	0.55	0.7614	0.38	0.8777	0.84	0.5597
IKKβ	3.08	**0.0389**	2.21	0.1036	0.46	0.8284	0.31	0.9228
**PI3K/AKT pathway**	***F* value**	***p* value**	***F* value**	***p* value**	***F* value**	***p* value**	***F* value**	***p* value**
SHIP2	2.79	**0.0531**	0.34	0.9026	0.20	0.9726	0.68	0.6702
p-eNOS (Ser^1177^)	9.34	**0.0003**	0.62	0.7132	0.16	0.9832	0.70	0.6513
p-AKT(Thr^308^)	13.68	**0.0000**	0.64	0.6990	0.85	0.5510	1.40	0.2816
p-AKT(Ser^473^)	1.60	0.2182	0.89	0.5267	1.15	0.3862	0.35	0.8953
PI3K p85	5.26	**0.0050**	0.44	0.8420	0.97	0.4816	1.10	0.4093
PI3K p100a	6.82	**0.0015**	0.69	0.6641	1.28	0.3288	0.54	0.7717
p-GSK3b (Ser^9^)	1.45	0.2652	1.21	0.3584	0.74	0.6252	0.77	0.6079
p-PTEN (Ser^380^)	0.72	0.6373	0.50	0.7977	1.15	0.3858	0.19	0.9732
**MAP kinase pathway**	***F* value**	***p* value**	***F* value**	***p* value**	***F* value**	***p* value**	***F* value**	***p* value**
p-HSP27(Ser^82^)	2.34	0.0891	1.77	0.1768	0.88	0.5331	1.17	0.3763
p-p38 MAPK (Thr^180^/Thr^182^)	0.70	0.6575	0.87	0.5386	1.04	0.4386	0.98	0.4743
**c-Jun pathway**	***F* value**	***p* value**	***F* value**	***p* value**	***F* value**	***p* value**	***F* value**	***p* value**
p-SAPK/JNK (Thr^183^/Tyr^185^)	1.60	0.2181	1.73	0.1870	0.60	0.7290	2.29	0.0946
MKK7	2.26	0.0982	1.32	0.3119	2.19	0.1065	0.29	0.9323
**ERK pathway**	***F* value**	***p* value**	***F* value**	***p* value**	***F* value**	***p* value**	***F* value**	***p* value**
MEK1/2	0.30	0.9267	0.47	0.8196	0.66	0.6799	0.44	0.8391
p-MEK1/2 (Ser^217^/Ser^221^)	0.85	0.5545	0.69	0.6590	0.15	0.9853	1.39	0.2838
ERK1/2	4.06	**0.0145**	2.53	0.0718	3.08	**0.0390**	0.10	0.9955
p-ERK1/2 (Thr^202^/Tyr^204^)	6.65	**0.0017**	2.41	0.0818	0.52	0.7850	0.74	0.6288
**SRC/JAK/STAT pathway**	***F* value**	***p* value**	***F* value**	***p* value**	***F* value**	***p* value**	***F* value**	***p* value**
p-STAT3 (Tyr^705^)	1.70	0.1932	1.36	0.2974	1.59	0.2207	0.40	0.8681
SOCS3	1.07	0.4255	0.23	0.9602	0.64	0.6938	0.43	0.8497
**IL-1b pathway**	***F* value**	***p* value**	***F* value**	***p* value**	***F* value**	***p* value**	***F* value**	***p* value**
TRAF6	4.15	**0.0133**	2.15	0.1122	1.67	0.2020	0.17	0.9820
p-TAK1 (Ser^412^)	1.67	0.2004	3.56	0.0237	0.43	0.8436	0.19	0.9752
**Inflammasome pathway**	***F* value**	***p* value**	***F* value**	***p* value**	***F* value**	***p* value**	***F* value**	***p* value**
p-STAT1(Tyr^701^)	1.75	0.1808	0.96	0.4874	1.32	0.3124	0.56	0.7530
**Apoptosis pathway**	***F* value**	***p* value**	***F* value**	***p* value**	***F* value**	***p* value**	***F* value**	***p* value**
BCL-2	1.40	0.2821	1.37	0.2913	0.71	0.6489	1.10	0.4077
p-BAD(Ser^136^)	3.08	**0.0390**	0.83	0.5673	5.33	**0.0047**	0.36	0.8930

The cells were cultured for three days in the absence or in the presence of 100 ng/mL EGF which was added daily. For any target the percentage changes in fluorescence intensity relative to untreated BxPC3 were calculated from three independent experiments. EGF pre-treated and non pre-treated BxPC3 and BxPC3-SMAD4+ cells were compared with each other by the analysis of variance (ANOVA) including in the model the effects of EGF, TGFβ1, S100A8/A9 alone or combined and of insulin.

### Chronic EGF stimulation enhances cell migration and MMP9 expression, not cell proliferation

**Figure 6:**
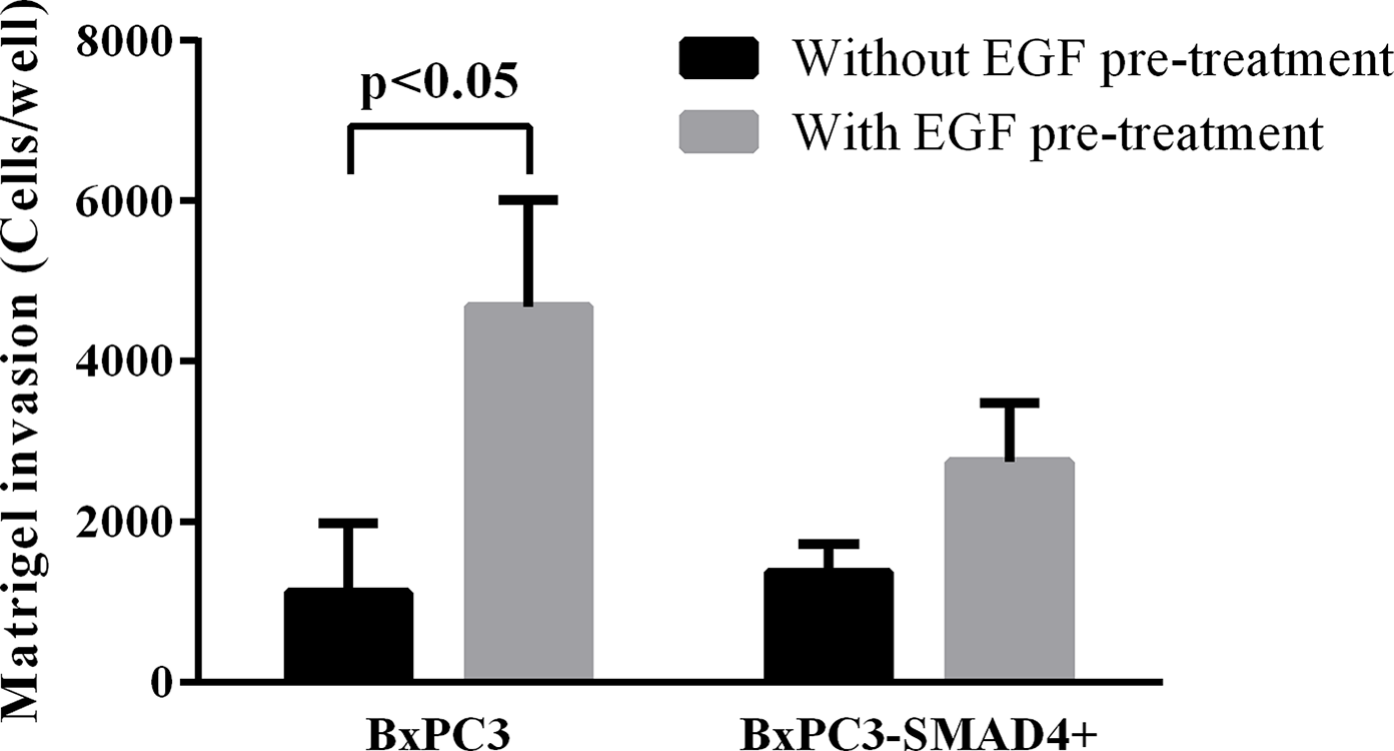
Matrigel invasion obtained from unstimulated and from EGF chronically stimulated pancreatic cancer cells expressing (BxPC3-SMAD4+) or not (BxPC3) SMAD4 Columns represent mean values, bars represent standard deviations obtained from four independent experiments each made in triplicate.

**Figure 7:**
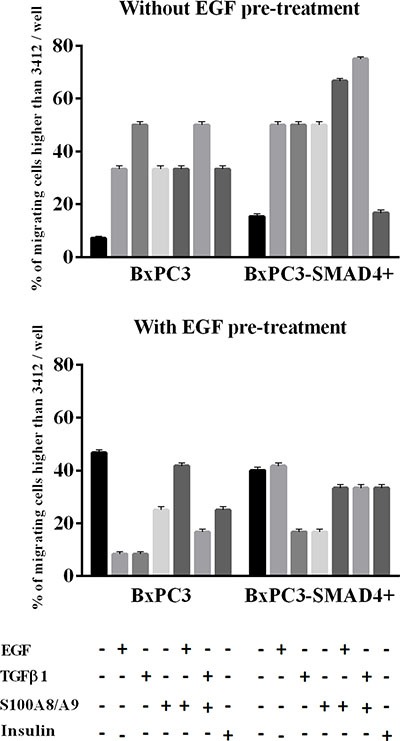
Matrigel invasion obtained from pancreatic cancer cells expressing (BxPC3-SMAD4+) or not (BxPC3) SMAD4 and subjected to insulin, EGF, TGFβ1 and S100A8/A9 stimulation in the absence or in the presence of chronic EGF exposure Columns represent the percentage of experiments with a number of migrating cells higher than 95^th^ percentile of their respective control cells. Four independent experiments, each made in triplicate, were performed resulting in 12 replicates for any studied condition.

Matrigel invasion assays were performed in order to verify whether the above-described alterations in cell signalling had an impact on cell biology. Chronic EGF stimulation significantly increased the number of migrating BxPC3-SMAD4+, but mainly BxPC3 cells (One-way ANOVA: *F* = 3.99, *p* = 0.0123) (Figure 6). The 95^th^ percentile value of untreated BxPC3 and BxPC3-SMAD4+ migrating cells was 3,412 cells/well. This value was used as a threshold to classify the number of migrating cells found in any experimental set as “comparable to” or “higher than” control cells. The percentage of BxPC3 and BxPC3-SMAD4+ experimental sets with a number of migrating cells higher than 3,412 cells/well is reported in Figure 7. In BxPC3-SMAD4+ cells, stimulation with S100A8/A9, EGF and TGFβ1 alone or combined caused a significant increase in migration (X^2^ = 15.4256, *p* = 0.017). In BxPC3 cells all stimuli increased the number of migrating cells with respect to control cells although not to a significant extent (X^2^ = 7.2738, *p* = 0.296). In EGF pre-treated BxPC3-SMAD4+ and BxPC3 cells, the studied stimuli neither enhanced nor reduced migrating cell numbers with the exception of EGF and TGFβ1 inhibitory effects in BxPC3 (X^2^ = 3.6008, *p* = 0.731 and X^2^ = 9.4574, *p* = 0.149, respectively).

Neither SMAD4 expression nor EGF chronic stimulation affected cell proliferation (*F* = 1.34, *p* = 0.2655). Only co-stimulation with S100A8/A9 and EGF caused a slight increase of BxPC3 cell proliferation (Supplementary Table 2).

**Figure 8:**
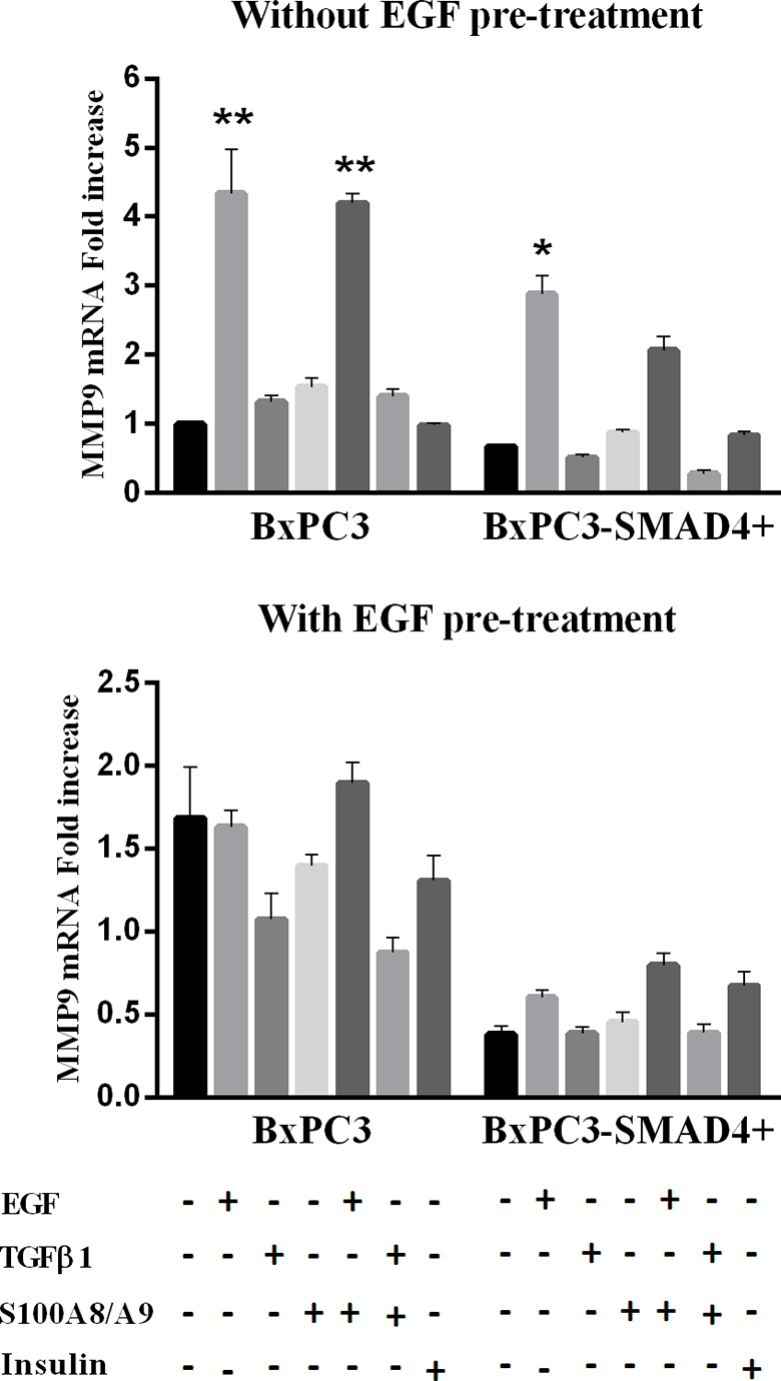
Matrix metalloproteinase 9 (MMP9) mRNA relative expression obtained from pancreatic cancer cells expressing (BxPC3-SMAD4+) or not (BxPC3) SMAD4 and subjected to insulin, EGF, TGFβ1 and S100A8/A9 stimulation in the absence or in the presence of chronic EGF exposure Columns represent mean values, bars represent standard errors obtained from three independent experiments each made in triplicate and report the relative mRNA fold increase with respect to BxPC3 without EGF pre-treatment. **= *p* < 0.01 with respect to unstimulated and TGFβ1 and S100A8/A9 stimulated BxPC3 cells; *= *p* < 0.05 with respect to unstimulated and TGFβ1 and S100A8/A9 stimulated BxPC3-SMAD4+ cells.

The relative expression of the metalloproteinases MMP8 and MMP9 was evaluated in the above-described conditions. Non-stimulated and stimulated BxPC3-SMAD4+ and BxPC3 cells did not express MMP8 (mean Ct > 40 cycles). Both BxPC3-SMAD4+ and BxPC3 cells expressed MMP9, which was significantly induced by a single acute stimulation with EGF alone or combined with S100A8/A9 in both cell lines (Two-way Anova: SMAD4 effect: *F* = 12.46, *p* < 0.0001; Treatment effect: *F* = 68.39, *p* < 0.0001; Interaction: *F* = 5.56, *p* = 0.114) (Figure 8). EGF pre-treatment caused in BxPC3 an increased (almost twice) and in BxPC3-SMAD4+ a decreased (almost half) MMP9 expression with respect to untreated BxPC3, and reduced in both cell lines the response to stimuli (Two-way Anova: SMAD4 effect: *F* = 55.52, *p* < 0.0001; Treatment effect: *F* = 13.92, *p* = 0.042; Interaction: *F* = 5.21, *p* = 0.470).

## DISCUSSION

EGFR overexpression is known to play a pivotal role in many cancer types including pancreatic carcinoma [36]. Based on this premise, several EGFR targeted therapies have been developed, and their use has been approved for the treatment of patients [37]. Yet these therapeutic approaches are often unsuccessful, particularly in pancreatic cancer patients [38]. This failure depends on several factors, including the complexity of the genetic alterations and tumor heterogeneity, the plasticity of tumor cells that enable them to activate alternative signalling pathways when one of them is antagonized, and the multiplicity of inflammatory molecules and growth factors from the stromal compartment, which might support EGFR-independent tumor cell growth and invasion [4, 20, 23, 39–41]. The aim of the present study was to improve our understanding of the way in which EGF governs the pancreatic cancer cell response to relevant stromal derived molecules, TGFβ1 and S100A8/A9, while also investigating whether SMAD4 participates in this complex scenario. The cellular model used in this study gave us the opportunity to investigate the role of SMAD4 mRNA, expressed by BxPC3-SMAD4+ but not by BxPC3 cells, independently from Smad4 protein, which was not expressed by any of our cell lines. Although it is unclear why SMAD4 translation did not occur in BxPC3-SMAD4+ cells, the results obtained in this study clearly indicate that SMAD4 transcripts regulate several SMAD4-related cellular patterns thus deciphering new roles for this relevant tumor suppressor gene. We first analyzed by RPPA a series of signalling pathways in pancreatic cancer cells, expressing or not expressing SMAD4, and repeatedly stimulated with EGF “*in vitro*” in order to simulate “*in vivo*” conditions. EGF chronic stimulation and SMAD4 were found to interact in the activation of ERK, MAP kinase and apoptosis pathways. In particular chronic exposure to EGF induced ERK and MAPK activation, and inhibited apoptosis in SMAD4 expressing cells [20]. EGF chronic stimulation exerted SMAD4-independent activation of SRC/JAK/STAT pathway and inhibition of the Wnt/β-catenin cascade. EGF chronic stimulation was also associated with an increased matrigel invasion, but not with an increased cellular proliferation in both SMAD4 expressing or not expressing cells in agreement with previous findings from Levy and Hill [42], thus supporting the hypothesis that EGF exacerbates pancreatic cancer progression and that therapeutic strategies aiming to block the EGF-EGFR axis are potentially beneficial. The increased tumor invasion is the end result of increased transcription of pro-survival and pro-invasive genes, including matrix metalloproteinases 8 and 9, which are believed to play a relevant role [43–45]. In the present study we demonstrated that MMP9, not MMP8, is induced by chronic EGF in SMAD4 non-expressing cells and inhibited in SMAD4 expressing cells. This highlights MMP9 as a potential target for further intervention at least in the subset of cancers carrying SMAD4 HD.

In the pancreatic cancer microenvironment, several tumor and stromal derived molecules target cancer cells, and cross-talk between the respectively activated pathways occurs [20]. A deleterious cross-talk between EGF and TGFβ pathways, enhanced by SMAD4 mutations, has been described by Deharvengt et al. [46], who studied a series of pancreatic cancer cell lines with a different SMAD4 profile. With respect to the above-cited study, our experimental model allows a clearer definition of the role of SMAD4 expression, the only one genetic difference between BxPC3 and BxPC3-SMAD4+ cells. TGFβ1 had only modest, if any, effect on cell signalling in SMAD4 expressing cells. By contrast, TGFβ1 caused a significant inhibition of NF-κB, PI3K/AKT and IL-1β pathways in the case of SMAD4 loss. These findings suggest that SMAD4 mRNA expression antagonizes TGFβ1-induced engagement of non-SMAD pathways, those most privileged being NF-κB, PI3K/AKT and IL-1β [26, 47, 48]. The cross-talk between EGF and TGFβ1 signalling was also supported by the results obtained in EGF pre-treated cells, which became almost insensitive to the inhibitory effects on cell signalling of TGFβ1 in a SMAD4-independent manner. Moreover, EGF and TGFβ join forces on cell migration: TGFβ1 enhanced cell migration independently from SMAD4 status in EGF non-pretreated cells, but had the opposite effect on EGF pre-treated cells. These findings appear in disagreement with those of Levy and Hill [42], who demonstrated that SMAD4 silencing completely abolished TGFβ1 induced migration and this discrepancy might depend on differences between the cellular models and TGFβ1 dosages. We used low amounts of TGFβ1 (0.02 ng/mL) because they were comparable to those used by Yasutome et al. [49], they were previously demonstrated by us to induce the epithelial to mesenchymal transition in BxPC3 [28] and they were consistent with the amount released by pancreatic tumor cells [50]. Although the low TGFβ1 dosage might be unable to induce Smad2/3 phosphorylation, never observed in our experimental conditions, a potential disruption in the TGFβ receptors might also be hypothesized [51]. The above observations regarding TGFβ1 could be translated to the heterocomplex S100A8/A9, since almost all the effects exerted by TGFβ1 on cell signalling, including the cross-talk with EGF signalling, and those on cell invasion were reproduced by the treatment of cells with S100A8/A9, thus confirming the existence of a biological similarity between TGFβ1 and S100A8/A9 as previously demonstrated by us in the context of epithelial to mesenchymal transition [28]. Interestingly, a potential anti-apoptosis effect through the induction of p-BAD [52], was obtained in case of SMAD4 not expressing cells when co-stimulated with both molecules and chronically exposed to EGF. This means that SMAD4 expression can prevent the activation of pro-survival pathways when cells are exposed to S100A8/A9 in the presence of TGFβ1 and EGF. From a mechanistic viewpoint, we suggest that SMAD4 HD, which correlates with tumor progression, creates a favorable ground for the activation of pro-survival pathways when multiple inflammatory stimuli, which are likely to occur in the tumor microenvironment, target cancer cells.

In the present study we also ascertained the effects of insulin, since it has been suggested that hyperinsulinemia due to insulin resistance is a risk factor for pancreatic cancer in patients with longstanding diabetes mellitus, while in those with early onset diabetes mellitus, it is considered a consequence of pancreatic cancer itself [53, 54]. Insulin did not influence any studied signaling pathways, but it favored matrigel invasion of BxPC3. This finding indicates that insulin probably accelerates pancreatic cancer progression in SMAD4 not expressing cells. Whether or not insulin is per se carcinogenetic is an open question.

It remains to be elucidated how SMAD4 mRNA, independently from Smad4 protein expression, might have a so relevant impact on cell signalling and cell migration. We verified whether SMAD4 expression caused differentially expressed proteins in the two studied cell lines. Among proteins underexpressed in BxPC3, we found Serum deprivation-response protein (SDPR), in line with findings by Fullerton et al. [55] who compared BxPC3 and BcPC3-p-INS4c5, a stable clone of BxPC3 expressing Smad4 protein, and showed SDPR mRNA as the most de-regulated gene. In BxPC3 we found a reduced expression of EGFR, this suggesting once again a link between the EGF and TGFβ pathways, and of Caveolin-1 (CAV1), which activates NF-κB [56], this being in line with the observed inhibition of this signalling pathway observed in this cell line. A role for differently expressed microRNA might also be hypothesized, since both canonical and non-canonical TGFβ signalling have been shown to be dependent on and to interact with several microRNA [57]. We focused our attention on miRNA 133a and miRNA 199, since they target the EGFR and Smad4 respectively [58, 59]. We observed that miRNA 133a expression was SMAD4 dependent, values being higher in BxPC3-SMAD4+ cells exposed or not exposed to chronic EGF (2.5 fold increase by RT-PCR), while miRNA 199 was both EGF and SMAD4 dependent, being induced by EGF chronic treatment in BxPC3 (3.3 fold increase by RT-PCR), not in BxPC3-SMAD4+ cells. This supports the hypothesis that SMAD4 expression might impact on signalling pathways like ERK through the de-regulation of microRNA [58].

In conclusion, SMAD4 homozygous deletion favors the constitutive activation of ERK, the EGF-induced activation of NF-κB and PI3K/AKT, and the EGF-induced inhibition of Wnt/β-catenin pathways, which are critical to cancer proliferation and metastases. TGFβ1 and S100A8/A9, which share their overall effects on pancreatic cancer cells, mainly inhibit NF-κB and PI3K/AKT in a SMAD4-dependent manner. The cross-talk between TGFβ1, S100A8/A9 and EGF signalling results in an overall de-sensitization of cancer cells to TGFβ1 and S100A8/A9 stimuli when chronically exposed to EGF, with the exception of the apoptosis pathway, the inhibition of BAD persisting in response to the combined action of TGFβ1 and S100A8/A9 in an SMAD-4 dependent manner. These pathways may be therefore potential targets for further therapeutic interventions.

## MATERIALS AND METHODS

### Cell lines

The pancreatic cancer cell line BxPC3 was kindly donated by Dr Andrea Galli (University of Florence, Italy). BxPC3-SMAD4+ cell line was obtained after clonal selection of BxPC3 cells transfected with the expression vector pBK-cytomegalovirus (CMV)-SMAD4/DPC4, as described by us elsewhere [28]. MiaPaCa2 were purchased by the American Type Culture Collection (Manassas, VA, USA) and Panc1 cells were donated by Prof Aldo Scarpa (University of Verona, Italy). MiaPaCa2 cells were cultured in DMEM while all the other cell lines were cultured in RPMI (Gibco, Life Technologies, Monza, Italy) supplemented with 10% fetal calf serum (FCS) (Gibco, Life Technologies, Monza, Italy), 2% L-Glutamine (MiaPaCa2) or 1% L-glutamine (all the other cell lines) (Gibco, Life Technologies, Monza, Italy) and 0.1% gentamycin (Gibco, Life Technologies, Monza, Italy).

### SMAD4 Real time (RT)-PCR

RT-PCR was used to verify the mRNA expression of SMAD4 gene. RNA was extracted from 1x10^6^ cells (MagnaPure Compact RNA isolation kit, Roche, Monza, Italy). One microgram of total RNA was reverse transcribed into cDNA (Random primers and Superscript TM II RNasiH-Reverse Trascriptase, Life Technologies, Monza, Italy). SMAD4 was PCR amplified (ABI Prism 7900 HT, Applied Biosystems, CA, USA) with 40 ng cDNA, 12.5 pmol/μL each of the primers pair 5′CCCAGGATCAGTAGGTGGAA3′ (Forward, exon 10) and 5′AAGGTTGTGGGTCTGCAATC3′ (Reverse exon 11), in a final volume of 20 μL containing MgCl 25 mM, dNTP 5 mM, EvaGreen 20× (Biotium, Inc., Hayward, CA, USA), SuperTaq 1 Unit (AB Analitica, Padova, Italy). After denaturation (5 minutes at 94°C), 30 amplification cycles (94°C for 30 seconds, 57°C for 30 seconds and 72°C for 30 seconds) were run. In all runs a positive control was always used (cDNA from 1x10^6^ MiaPaca2 and PANC cell lines). Each sample was analyzed in duplicate.

### SILAC experiment (Cell culture, In-gel digestion, LC-MS/MS and data analysis)

In a first experiment, BxPC3 and BxPC3-SMAD4+ cell lines were cultured in RPMI 1640 MEDIA FOR SILAC with 10% dialyzed fetal bovine serum (FBS), additioned either with the non-labelled aminoacids Lysin and Arginine (light medium) or with the labelled ^13^C_6_-Lysine and ^13^C_6_^15^N_4_-arginine (heavy medium) (Chemical Research 2000 srl, Rome, Italy). In a second experiment, the same cell lines were cultured by swapping media, being the BxPC3 maintained in heavy medium and BxPC3-SMAD4+ maintained in light medium, creating two biological replicates of the same experiment. After 8 days, cell media were changed with freshly media prepared as specified above excluding the serum addition, to reduce the possible amount of contaminant in proteomic analyses.

Ten μg of proteins of each biological replicate were loaded onto a precast gel (NuPAGE, 4–12% Bis-Tris, Life Technologies, Monza, Italy) and electrophoresis was carried out. Each lane was then divided into 4 slices which were then subjected to reduction/alkylation and in-gel digestion with sequencing grade modified trypsin (Promega, Madison, WI, USA) as previously described [60]. Peptides were extracted from the gel by 3 changes of 50% acetonitrile/0.1% formic acid (FA). Samples were dried under vacuum, suspended in 3% acetonitrile/0.1% FA and loaded into a 10 cm pico-frit column (75 um I.D., 15 um Tip, New Objective, Woburn, MA, USA) packed in-house with C18 material (Aeris Peptide 3.6 um XB-C18, Phenomenex, Bologna, Italy). Peptides were separated with a HPLC Ultimate 3000 (Dionex – Thermo Fisher Scientific, Waltham, MA, USA) using a linear gradient from 3 to 50% of acetonitrile/0.1 FA in 90 min at a flow rate of 250 nL/min. LC-MS/MS analysis was conducted with a LTQ-Orbitrap XL mass spectrometer (Thermo Fisher Scientific). Data were analyzed with the Proteome Discoverer software (version 1.4, Thermo Fisher Scientific) interfaced to a Mascot server (version 2.2.4, Matrix Science, London, UK) and searched against the human section of the Uniprot Database (www.uniprot.org, version 20150401, 90411 sequences) using carbamidomethyl cysteine as static modification and ^13^C_6_-Lysine, ^13^C_6_^15^N_4_-arginine, and methionine oxidation as variable modifications. Precursor and fragment tolerance were set at 10 ppm and 0.6 Da, respectively. Samples were searched using a MudPIT protocol and the algorithm Percolator was used to calculate False Discovery Rate (FDR) based on the search against a randomized database. The results were filtered in order to consider only proteins identified with at least two unique peptides and high confidence (*q* < 0.01). Only unique peptides were considered for quantification. The ratio between light/heavy and heavy/light were calculated for each identified protein of the two experiments. Proteins were considered as significantly altered if the average value, calculated for the two ratios, was either above 1.5 or less than 0.67.

### Experimental design

BxPC3 and BxPC3-SMAD4+ were cultured for 3 days in the absence and in the presence of 100 ng/mL EGF (ProSpec-Tany TechnoGene Ltd., D.B.A. Italia, Segrate, Italy). Fresh media with or without EGF were daily replaced. On the fourth day (experimental day) the cells were not stimulated (negative control) or were stimulated with 100 ng/mL EGF, 0,02 ng/mL TGFβ1 (ProSpec-Tany TechnoGene Ltd., D.B.A. Italia, Segrate, Italy), 10 nM S100A8/A9 (ProSpec-Tany TechnoGene Ltd., D.B.A. Italia, Segrate, Italy), or with 50 mU insulin (positive control) (Insuman Rapid, Sanofi-Aventis, Milano, Italy). For RPPA and immunoblot analyses the cells were collected ten minutes after stimulation. For Matrigel invasion assay and matrix metalloproteinases expression the cells were collected 24 hours after stimulation. Cell proliferation was evaluated 72 hours after stimulation.

### Reverse phase protein array (RPPA) analysis

RPPA analysis was performed using a procedure previously optimized and validated [61]. Briefly, cell lysates obtained from 800,000 cells (ø 10 cm Petri dishes) were solubilized in 4×SDS loading buffer (Sigma-Aldrich, Milano, Italy) and heated for 5 minutes at 95°C. Samples were spotted in duplicates onto nitrocellulose-coated glass slides (Grace Bio-labs, Bend, OR, USA) using a microarraying robot (MicroGrid 610, Digilab, Marlborough, MA, USA). The printed slides were blocked and incubated overnight at 4°C with shaking with the specific primary antibodies (Supplementary Table 3) (Cell Signalling Technology, Danvers, MA, USA). β-actin was included as a house-keeping protein to control protein loading. After incubation with infrared secondary antibodies (800 CW LI-COR anti-rabbit antibody and 700 CW LI-COR anti-mouse antibody), the slides were scanned with a Licor Odyssey scanner (LI-COR, Biosciences, Lincoln, NE, USA) at 21 μm resolution at 700 and 800 nm. The fluorescent data were processed with GenePix Pro-6 Microarray Image Analysis software (Molecular Services Inc., Sunnyvale, CA, USA). Protein signals were determined with background subtraction and normalization to the internal housekeeping targets using an RPP analyzer.

### Immunoblot analysis

For immunoblot analysis, 800,000 cells were used (ø 10 cm Petri dishes). Petri dishes were transferred into an ice bath, and the cells were washed twice with cold PBS, and re-suspended in 100 μL of cold lysis buffer [20 mM Tris–HCl, pH 7.5, 150 mM NaCl, 1 mM EDTA, 1% Triton-X 100, 50 mM NaF, 10 mM Na_4_P_2_O_7_,1 mM Na_3_VO_4_, and 10% protease inhibitor cocktail (Sigma Aldrich SRL, Milano, Italy)]. Lysates were centrifuged for 10 minutes at 14,000 rpm at 4°C, and total proteins in the supernatants were measured using the Bio-Rad protein assay (Bio-Rad Laboratories, Milano, Italy). For each sample, 40 μg proteins were electrophoresed through 4–12% NuPAGE^®^ Novex Bis-Tris SDS–PAGE Gel or 3–8% NuPAGE^®^Novex Tris-Acetate SDS–PAGE Gel (Life Technologies, Monza, Italy) and electrophoretically transferred to Nitrocellulose Membrane (iBlot^®^ Transfer Stack, Life Technologies, Monza, Italy) by means of the iBlotTM Dry Blotting System (Life Technologies, Monza, Italy). Following incubation for 1 hour in a blocking buffer [5% low fat powder milk re-suspended in PBS-T (PBS with 0.1% Tween-20)], membranes were incubated overnight at 4°C with the primary antibodies [anti-Smad4, anti-phospho-Smad2 (Ser^465/467^), anti-Smad2, anti-phospho-Smad3 (Ser^423/425^), anti-Smad3, anti-phospho-Akt (Ser^473^, Thr^308^), anti-Akt, anti-β-actin, anti-phospho-mTOR (Ser^2448^, Ser^2481^), anti-phospho-β catenin (Ser^33/37^/Thr^41^), anti-β catenin, anti-phospho-p44/42 MAPK (Erk1/2)(Thr^202^/Tyr^204^) (Cell Signalling Technology, Danvers, MA, USA); anti-phospho IkB-α (Ser^32^) (Santa Cruz Biotechnology Inc., Santa Cruz, CA, USA)], diluted 1:5000 (β-actin), 1:2000 (mTOR) or 1:3000 (all the others) in the blocking buffer. The blots, washed three times in PBS-T for 15 minutes each time, were incubated with alkaline phosphatase-conjugated anti-rabbit (Cell Signalling Technology, Danvers, MA, USA) or anti-goat (Sigma-Aldrich, Milano, Italy) secondary antibodies and then washed three times in PBS-T for 15 minutes each time and developed with the ECL Advance Western Blot Detection Kit (GE Healthcare Technologies, Milan, Italy). Each experiment was performed at least in triplicate.

Nuclear proteins were extracted by the “NE-PER nuclear and cytoplasmic extraction reagents” (Thermo Scientific, Rockford, IL, USA) following the manufacturer's instructions. Immunoblot analysis of nuclear extracts were performed in the same conditions described above.

### Matrigel invasion assay

For matrigel invasion experiments twelve-well plates (ø 12 mm) with polycarbonate membrane filters (Transwell, Corning Costar Corporation, Milano, Italy) were used. Matrigel (BD Biosciences, Bedford, MA, USA), diluted with cold RPMI to the desired final concentration (1.5 μg/μL), was applied (200 μL) to the filters and dried overnight in a humidified atmosphere at 37 °C. For each condition, 100 x 10^3^ cells, suspended in 500 μL RPMI containing 0.1% bovine serum albumin (BSA) (Sigma-Aldrich, Milano, Italy), were loaded on the Matrigel layer (upper chamber); corresponding tumor conditioned RPMI (700 μL) was added to the lower chamber. The cells were then incubated for 48 hours in a humidified atmosphere at 37 °C. After Matrigel removal, each filter was cut and transferred onto a new well of a new plate containing 100 μL Luciferase (Cell Titer-Glo® Luminescent Cell Viability Assay, Promega, Madison, WI, USA), which allows the detection of viable cells. After 10 minutes' incubation at room temperature in the dark, luminescence was measured (counts per second, cps) using the multilabel counter Victor3 (Perkin Elmer, Waltham, MA, USA). For each condition a series of at least three separate experiments, each made in duplicate, were performed. In each experiment serial dilutions of a known number of BxPC3 and of BxPC3-SMAD4+ cells were prepared and analysed as described above to obtain a correlation between the number of cells and cps. The regression line obtained was used to calculate the number of migrating cells based on cps results.

### Cell proliferation XTT assay

Cell growth was assessed using the XTT cell viability test (Roche Diagnostics S.p.A., Monza, Italy). Briefly, 2,000 cells per well were seeded in 96-well cell culture plates and treated as detailed in the experimental design. After 72 hours' stimulation, 100 μL XTT reagent was added to each well, and left for for 4 hours before Abs_450nm_ analysis (PR 3100 TSC, Bio-Rad Laboratories, Milano, Italy).

### MMP8 and MMP9 mRNA expression analyses

For expression analyses 150,000 cells seeded in six-well plates were used. Total RNA was isolated (High Pure RNA Isolation Kit, Roche Diagnostics S.p.A., Monza, Italy) according to the manufacturer's instructions. Three μg of total RNA was reverse transcribed into cDNA (Random primers and Superscript II -Reverse Trascriptase, Life Technologies, Monza, Italy). The relative quantification of MMP8 and MMP9 mRNA was undertaken by RT-PCR with an ABI Prism 7900 HT (Applied Biosystems, CA, USA). Primers (MMP8-F: 5′CACTCCCTCAAGATGACATCGA3′-R:5′ACGGAGTGTGGTGATAGCATCA3′; MMP9-F: 5′CCTGGGCAGATTCCAAACCT3′- R: 5′GCAAGTCTT CCGAGTAGTTTTGGAT3′) and fluorogenic probes (MMP8-5′FAM-CAAGCAACCCTATCCAACCTACTGG ACCAA-TAMRA3′; MMP9-5′FAM-CTCAAGTGGCACC ACCACAACATCACC-MGB3′) for relative quantifications were performed in a final volume of 20 μL for MMP8 and MMP9. For each reaction, 150 ng cDNA, 200 nM probe and 500 nM (MMP8/MMP9) primers were used. The reference gene, HPRT1, was selected according to the method commonly used for internal control for quantitative gene expression analyses, and its expression was determined using commercially available HPRT1 primers and probe sets (PDARs part number 4326321E, Applied Biosystems, CA, USA). MMP8, MMP9 and HPRT1 were analyzed in triplicate for each sample. MMP8 and MMP9 PCR reactions were 2 minutes at 50°C and 10 minutes at 95°C respectively, followed by 40 cycles of 15 seconds at 95°C and 1 minute at 60°C. To determine the relative RNA of target genes levels we used the comparative Ct method, a mathematical model that calculates changes in gene expression as a relative fold difference between an experimental and a calibrator sample.

### Statistical analysis

The statistical analysis of data was made by the Analysis of variance, Bonferroni's test for pairwise comparisons and the chi-square test using Stata Ver. 13.1 (StataCorp, Texas, USA).

## SUPPLEMENTARY MATERIALS




