# Supplementary material for: SMAD4 loss enables EGF, TGFβ1 and S100A8/A9 induced activation of critical pathways to invasion in human pancreatic adenocarcinoma cells
Source: Oncotarget. 2016 Sep 16;7(43):69927–44. doi: 10.18632/oncotarget.12068 (PMC5342525; doi:10.18632/oncotarget.12068)
Supplement: Supplementary file 1 [file oncotarget-07-69927-s001.pdf]

# SMAD4 loss enables EGF, TGFβ1 and S100A8/A9 induced activation of critical pathways to invasion in human pancreatic adenocarcinoma cells

## Supplementary Materials

**Supplementary Table S1: Expression of BxPC3 and BxPC3-SMAD4+ cellular proteins compared by means of SILAC experiments (two independent experiments).** A total of 1476 and 1102 proteins were identified by means of Proteome Discoverer software. The results of the two experiments were matched and averaged, this resulting in a total of 1002 proteins here reported. A significant differential expression was considered when the ratio between BxPC3 and BxPC3-SMAD4+ for any protein was below 0.67 (underexpressed in BxPC3) or above 1.5 (overexpressed in BxPC3). See Supplementary_Table_1

**Supplementary Table S2: Cell proliferation**

| | BxPC3 | BxPC3-SMAD4+ | BxPC3 | BxPC3-SMAD4+ |
|---|---|---|---|---|
| EGF pre-treatment | No | No | Yes | Yes |
| Control | 99.02 ± 12.65 | 101.02 ± 16.22 | 91.77 ± 19.10 | 97.02 ± 18.48 |
| EGF | 103.53 ± 16.12 | 107.98 ± 23.16 | 92.02 ± 22.52 | 96.42 ± 15.07 |
| TGFβ1 | 98.02 ± 23.69 | 106.37 ± 26.82 | 94.64 ± 14.52 | 101.35 ± 16.59 |
| S100A8/A9 | 106.81 ± 9.65 | 104.03 ± 24.95 | 85.70 ± 24.26 | 98.89 ± 18.99 |
| S100A8/A9+EGF | 113.89 ± 18.93* | 110.90 ± 23.14 | 90.93 ± 31.87 | 97.61 ± 20.27 |
| S100A8/A9+TGFβ1 | 112.04 ± 10.32 | 108.65 ± 22.26 | 79.38 ± 17.04 | 98.99 ± 19.05 |
| Insulin | 100.46 ± 13.02 | 91.90 ± 20.64 | 93.57 ± 15.22 | 94.35 ± 20.84 |
| | $F = 3.92$ $p = 0.0011$ | $F = 1.91$ $p = 0.0828$ | $F = 1.51$ $p = 0.177$ | $F = 0.34$ $p = 0.9124$ |

*$p < 0.05$ with respect to control.

A total of 12 experiments were performed, each made in duplicate. Findings from stimulated cells were expressed as percentage with respect to median values of the respective controls.

**Supplementary Table S3: List of primary antibodies used in Reversed Phase Protein Array (RPPA) signalling study**

| **TNF receptor 1 pathway** | |
|---|---|
| **Antibody** | **dilution** |
| A20/TNFAIP3 | 1:150 |
| TRAF2 | 1:500 |
| p-RIP2(Ser$^{176}$) | 1:500 |
| **NF-κB pathway** | |
| IKKα | 1:250 |
| IKKβ | 1:250 |
| p-IKKα/β(Ser$^{176}$/Ser$^{177}$) | 1:250 |
| p-NF-κB p65(Ser$^{536}$) | 1:100 |
| **PI3K/AKT pathway** | |
| SHIP2 | 1:2,000 |
| p-eNOS(Ser$^{1177}$) | 1:500 |
| p-AKT(Thr$^{308}$) | 1:50 |
| p-AKT(Ser$^{473}$) | 1:25 |
| PI3K p100a | 1:250 |
| PI3K p85 | 1:250 |
| p-GSK3β(Ser$^{9}$) | 1:500 |
| p-PTEN(Ser$^{380}$) | 1: 1,000 |
| **MAP kinase pathway** | |
| p-p38 MAPK(Thr$^{180}$/Tyr$^{182}$) | 1:1,000 |
| p-HSP27(Ser$^{82}$) | 1:50 |
| **c-Jun pathway** | |
| p-SAPK/JNK(Thr$^{183}$/Tyr$^{185}$) | 1:1,000 |
| MKK7 | 1:100 |
| **ERK pathway** | |
| MEK1/2 | 1:250 |
| p-MEK1/2(Ser$^{217}$/Ser$^{221}$) | 1:250 |
| p-ERK1/2(Thr$^{202}$/Tyr$^{204}$) | 1:2,000 |
| ERK1/2 | 1:100 |
| **SRC/JAK/STAT pathway** | |
| p-STAT3(Tyr$^{705}$) | 1:100 |
| SOCS3 | 1:500 |
| **IL1β pathway** | |
| TRAF6 | 1:500 |
| p-TAK1(Ser$^{412}$) | 1:100 |
| **Inflammasome pathway** | |
| p-STAT1 (Tyr$^{701}$) | 1:250 |
| **Apoptosis pathway** | |
| BCL-2 | 1:250 |
| p-BAD(Ser$^{136}$) | 1:500 |

All antibodies were rabbit antibodes and were purchased from Cell Signalling Technology (Cell Signalling Technology, Danvers, MA, USA).

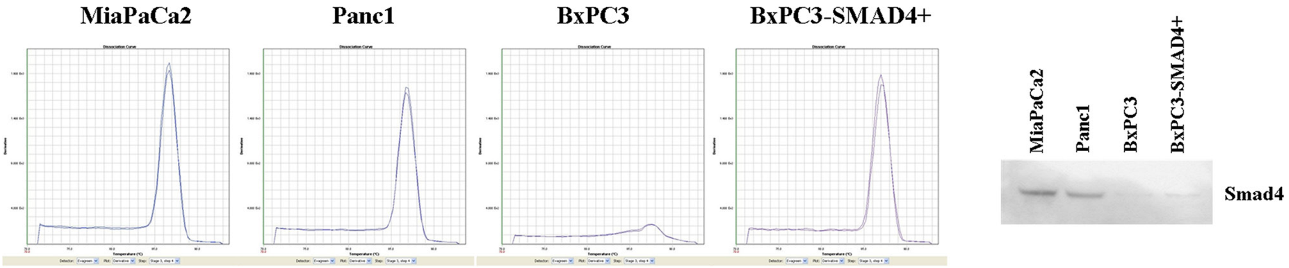


**Supplementary Figure S1: SMAD4 mRNA and protein expression.** Melting curves (left panels) obtained from the analysis of two cell lines without SMAD4 HD (MiaPaCa2 and Panc1), from BxPC3 and BxPC3-SMAD4+ cells. Western blot analysis of Smad4 from the same cell lines is shown in the right panel.

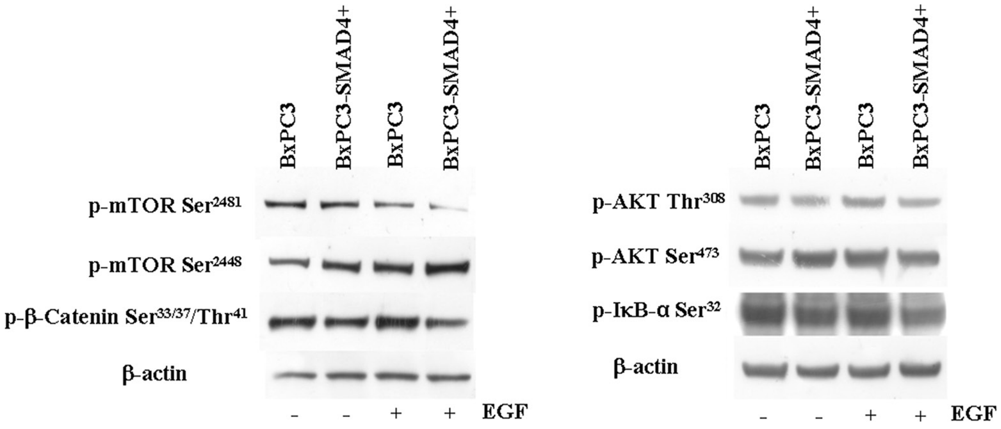


**Supplementary Figure S2: Western blot analyses of p-mTOR (Ser2481 and Ser2448), p-β-catenin (Ser33/37/Thr41) and of β-actin obtained from unstimulated and from EGF chronically stimulated pancreatic cancer cells expressing (BxPC3-SMAD4+) or not (BxPC3) SMAD4.** The cells were cultured for three days in the absence (−) or in the presence (+) of 100 ng/mL EGF which was added daily.

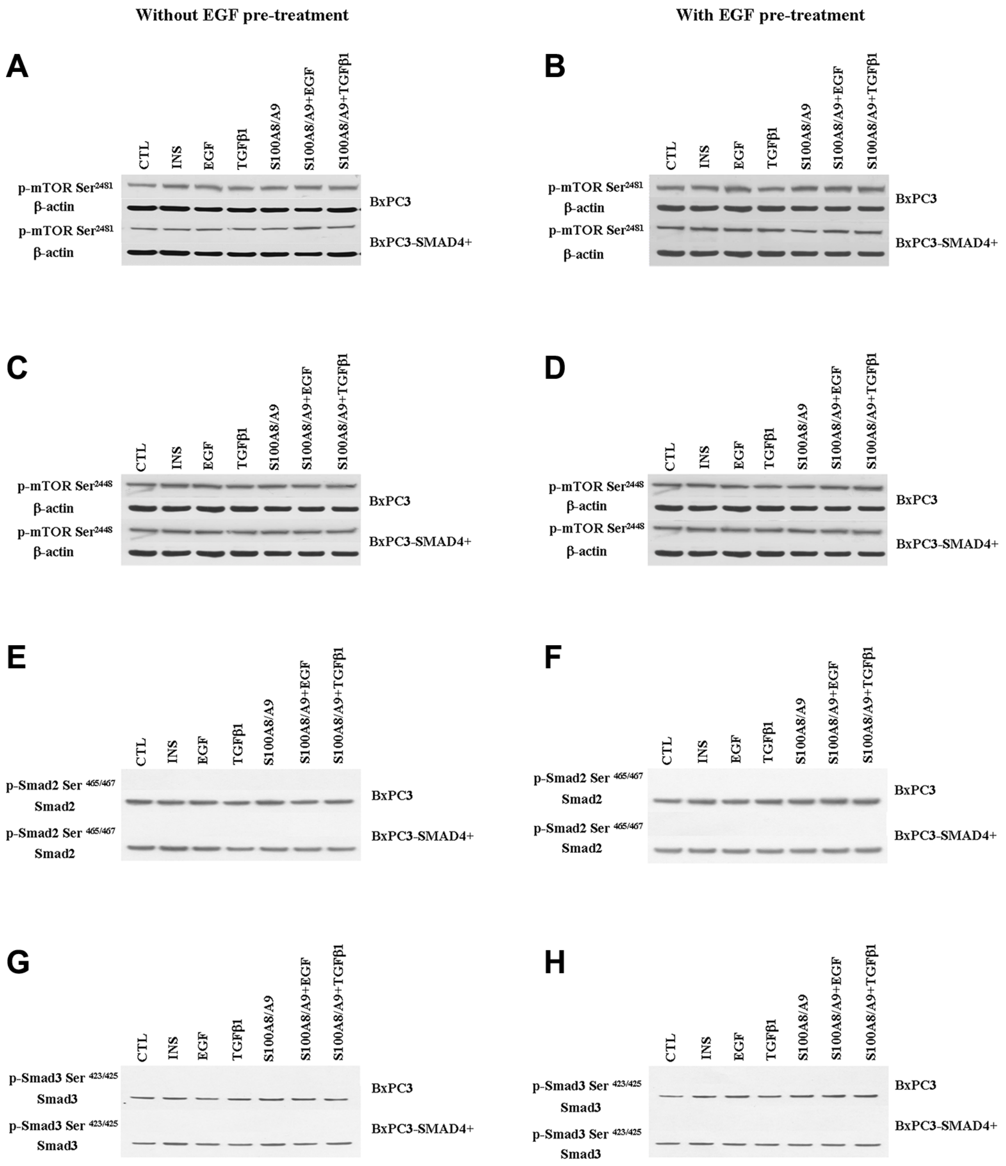


**Supplementary Figure S3: Western blot analyses obtained from pancreatic cancer cells expressing (BxPC3-SMAD4+) or not (BxPC3) SMAD4 and subjected to insulin (INS), EGF, TGFβ1 and S100A8/A9 stimulation in the absence or in the presence of chronic EGF exposure.** Representative targets of the mTOR (panels **A**, **B**, **C** and **D**) pathways are shown. Phosphorylated and not-phosphorylated Smad2 and Smad3 are also shown (panels **E**, **F**, **G** and **H**).
